# Noninvasive biomarkers for the diagnosis of steatohepatitis and advanced fibrosis in NAFLD

**DOI:** 10.1186/2050-7771-1-7

**Published:** 2013-02-04

**Authors:** Steven G Pearce, Nirav C Thosani, Jen-Jung Pan

**Affiliations:** 1 Division of Gastroenterology, Hepatology and Nutrition, Department of Internal Medicine, The University of Texas Health Science Center at Houston, 6431 Fannin Street, MSB 4.234, Houston, TX 77030, USA

**Keywords:** Biomarker, Noninvasive, NAFLD, NASH, Fibrosis

## Abstract

Nonalcoholic fatty liver disease (NAFLD) is the most common cause of abnormal liver enzymes in both adults and children. NAFLD has a histologic spectrum ranging from simple steatosis to nonalcoholic steatohepatitis (NASH), advanced fibrosis, and cirrhosis. It is imperative to distinguish simple steatosis from NASH since the latter has a progressive disease course and can lead to end-stage liver disease. Liver biopsy has been considered as the gold standard for the diagnosis of NASH. However, liver biopsy is invasive, costly, and can rarely cause significant morbidity (risk of morbidity, 0.06-0.35%; risk of mortality, 0.1-0.01%). Imaging studies such as ultrasonography, computed tomography, and magnetic resonance imaging have limited sensitivity in detecting steatosis and cannot distinguish steatosis from NASH. Alanine aminotransferase (ALT) has been used as a surrogate marker for liver injuries. However, ALT is not an ideal marker for either diagnosis of NAFLD or distinguishing steatosis from NASH. Better noninvasive biomarkers or panels of biomarkers that are cheaper, reliable, and reproducible are urgently needed for patients with NASH to assist in establishing diagnosis, providing risk information, and monitoring disease progression and treatment response. In this article, we plan to concisely review the current advances in the use of biomarkers for the diagnosis of NASH.

## Introduction

Nonalcoholic fatty liver disease (NAFLD) is the most common cause of abnormal liver function test results in both adults and children [1,2]. NAFLD in fact covers a histological spectrum ranging from simple steatosis to nonalcoholic steatohepatitis (NASH), advanced fibrosis, and cirrhosis [1]. Simple steatosis without fibrosis or inflammation has a benign clinical course in most but not in all cases without excess mortality [3]. NASH, on the other hand, may have a more progressive course that can lead to cirrhosis in 10-15% of patients [4]. Survival is lower in patients with NASH based on the findings from long-term longitudinal studies [3,5]. It is therefore imperative to distinguish simple steatosis from NASH in order to provide risk stratification and intervention slowing down disease progression for patients with the latter condition.

Liver biopsy remains the gold standard for making the diagnosis of NASH. However, this procedure is invasive, costly, and is associated with rare but potential complications and sampling errors. Hence it is not suitable as a screening tool for a condition that affects one-third of the American population [6]. Imaging studies such as ultrasonography, computed tomography (CT), and magnetic resonance imaging have been used to diagnose NAFLD. These modalities have the advantages of being noninvasive and can be repetitively performed over a period of time. Nevertheless, none of them have sufficient sensitivity and specificity for staging the disease and cannot distinguish between simple steatosis and NASH with or without fibrosis [6].

Alanine aminotransferase (ALT) has been used as a surrogate marker for liver injury. Several studies suggested that ALT is not an ideal biomarker for either diagnosis of NAFLD or distinguishing simple steatosis from NASH. In a cross-sectional population study, examining hepatic steatosis based on the measurement of hepatic triglyceride content by magnetic resonance spectroscopy, nearly one-third (31%) of the subjects had hepatic steatosis. Most of the subjects with steatosis (79%) had normal serum ALT levels [7]. In another study, 58 of 80 (72.5%) patients who had a normal ALT and underwent surgery unrelated to liver diseases had varying degrees of NASH. Among those patients, 26 had fibrosis and 8 had silent cirrhosis [8]. The third study reported that the entire histologic spectrum of NAFLD could be seen in the individuals with normal ALT values. The histological spectrum of NAFLD in people with NAFLD and normal ALT was not significantly different from those with NAFLD and elevated ALT. A low normal ALT value therefore does not guarantee freedom from underlying NASH with advanced fibrosis [9].

As mentioned earlier, the current available tests have significant limitations in distinguishing simple steatosis from NASH, which has important clinical implications. Better noninvasive biomarkers or panels of biomarkers that are cheaper, reliable, and reproducible are urgently needed for patients with NASH to assist in establishing diagnosis, providing risk information, and monitoring disease progression and treatment response. In this article, we plan to concisely review the current advances in the use of biomarkers for the diagnosis of NASH.

### Proinflammatory markers and NASH

NAFLD is considered to be the hepatic manifestation of metabolic syndrome (MetS) [10]. Obesity, a common feature of NAFLD and MetS, is associated with a chronic and subacute inflammatory state that is both systemic and focally localized in certain tissues such as liver. Consistent with the potential roles of inflammation, serum levels of proinflammatory cytokines are elevated in obese subjects [11]. NASH is a progressive form of NAFLD where in addition to simple steatosis, inflammation, hepatocyte ballooning, and sometimes, fibrosis are observed [12]. It is therefore of great interest to determine if NASH can be distinguished from simple steatosis by using proinflammatory biomarkers. Below we will discuss the current findings of the use of proinflammatory biomarkers for the diagnosis of NASH.

#### Tumor necrosis factor alpha (TNF-α)

TNF-α plays an important role in insulin resistance, the pathognomonic feature of MetS, through inhibiting the tyrosine kinase activity of the insulin receptor [13]. Abiru et al. reported that patients with NASH had significantly higher serum TNF-α and its soluble receptor (sTNFR1) than those with simple steatosis, although they did not provide a cutoff value of the cytokine for clinical use [14]. A recent study further reported that patients with NASH had higher levels of TNF-α messenger ribonucleic acid (mRNA) than healthy controls. The authors proposed a TNF-α mRNA cutoff value of 100 ng/mL predicted NASH [area under receiver operating characteristic curves (AUROC) 0.685, sensitivity 66.7%, specificity 74.1%] [15]. The role of TNF-α in NASH is further supported by the beneficial effects of pentoxifylline, an antagonizer of TNF-α, on biochemical and histological activity associated with NASH [16-18].

#### Interleukin-6 (IL-6)

IL-6 is implicated in insulin resistance, at least in part, through the induction of suppressor of cytokine signaling-3 in liver [19]. Several studies have reported a strong association between IL-6 and NASH. In a pilot study with a small cohort, patients with NASH received special diet plus exercise with or without antioxidant vitamin E (800 IU/day for 6 weeks). Plasma IL-6 levels were significantly higher in patients with NASH and the levels decreased with the therapy [20]. Not only the cytokine itself, but its soluble receptor is also significantly increased in patients with NASH than those with simple steatosis [14]. No cutoff value of the cytokine for differentiating NASH from simple steatosis was provided from either one of the studies. In the third small cohort study, morbidly obese patients were divided into 3 groups based on the histologic findings: non-NASH, probable NASH, and NASH. IL-6 level was correlated with the degree of steatosis until the patients met the criteria of NASH when their blood IL-6 levels decreased. Multivariate logistic regression analysis identified the level of IL-6 > 4.81 pg/mL [odds ratio (OR): 33.7, 95% confidence interval (CI): 1.7-680.7, p ≤ 0.022] as an independent predictor of the degree of steatosis but not of NASH [21]. In the fourth study, NASH could be well distinguished from simple steatosis when using the cutoff value of IL-6 at 4.6 pg/mL (AUROC 0.817, sensitivity 58.1%, specificity 100%). The authors concluded that IL-6 was highly specific in confirming the absence of NASH at normal values [22].

#### C-reactive protein (CRP)

CRP levels were elevated in NAFLD patients compared with controls matched by age and body mass index (BMI) and hence were reported to be an independent risk factor for NAFLD [23]. However, the study was limited by the lack of histologic diagnosis since NAFLD was diagnosed based on elevated ALT and sonographic evidence of fatty liver. It is still controversial whether CRP can differentiate NASH from simple steatosis. For example, high-sensitivity CRP (hs-CRP) levels were significantly higher in patients with NASH compared with those with steatosis in a Japanese study [24]. In addition, hs-CRP levels were significantly elevated in patients with NASH and advanced fibrosis compared with those with NASH and mild fibrosis. However, there was no relation between serum hs-CRP levels and either hepatic steatosis or necroinflammation grade. The AUROC for distinguishing between NASH and steatosis using hs-CRP was 0.833 [24]. In a Romanian study, CRP had an excellent performance in predicting the presence of NASH using a cutoff value of 3.5 mg/L (AUROC 0.906, sensitivity 82%, specificity 88%). CRP, however, was not a predictor of severe fibrosis (F ≥ 3) in that study [25].

In contrast, a small cohort study (18 patients with NASH) reported that there was no correlation of CRP levels with either the degree of hepatic steatosis, inflammation, or the stage of liver fibrosis despite the fact that NASH patients had significantly higher CRP levels than 16 controls [26]. Similar to the previous study, an Australian group reported that patients with NASH and simple steatosis had similar hs-CRP levels. No relationship existed between hs-CRP levels and the grades of hepatic steatosis, necroinflammation, and fibrosis [27]. Both studies are underpowered due to inadequate sample sizes.

CRP may be a marker of hepatic steatosis but not of severity of NAFLD in obese patients [28].

#### Pentraxin 3 (PTX3)

PTX3 is a prototypic member of the long chain pentraxin family and a newly discovered marker of the acute phase inflammatory response. PTX3 is structurally related to, but distinct from classic members of the short chain pentraxin family, including CRP [29]. Yoneda et al. [29] first reported that the plasma PTX3 level was significantly higher in patients with NASH than those with simple steatosis or healthy controls. No significant difference of plasma PTX3 levels was observed between the simple steatosis and control groups. A stepwise increase in plasma PTX3 levels was found as the stages of hepatic fibrosis increased. PTX3, however, was not related to either hepatic steatosis or necroinflammation grade. Using 1.61 ng/mL as a cutoff value, PTX3 had a fair performance in differentiating NASH from simple steatosis (AUROC 0.755, sensitivity 66.7%, specificity 78.6%). Using 2.45 ng/mL as a cutoff value, PTX3 had a good performance in distinguishing stage 3–4 from stage 0–2 NAFLD (AUROC 0.850, sensitivity 70.6%, specificity 94.3%) [29].

#### Ferritin

Increased ferritin but normal transferrin saturation is frequently found in patients with hepatic steatosis. The simultaneous disorder of iron and glucose and/or lipid metabolism, in most of the cases associated with insulin resistance, is responsible for persistent hyperferritinemia and identifies patients at risk for NASH [30]. Indeed, serum ferritin level was significantly higher in the NASH patients than those with simple steatosis, according to a Japanese study [31]. In that study, the serum ferritin level was related with insulin resistance. The performance of serum ferritin for distinguishing NASH from simple steatosis was fair (AUROC 0.732) and the optimal cutoff value was 196 ng/mL with a sensitivity 64.2% and specificity 76.5%. In another study, the performance of serum ferritin for detecting NASH was improved (AUROC 0.82) when using a higher cutoff value 240 ng/mL with the price of losing specificity (sensitivity 91%, specificity 70%) [32]. In a recent large cohort study using gender-specific cutoff values (> 300 ng/mL in women and > 450 ng/mL for men), serum ferritin levels greater than 1.5 times of upper limit of normal was associated with hepatic iron deposition, a diagnosis of NASH, and worsened histologic activity, and is an independent predictor of advanced hepatic fibrosis among patients with NAFLD. The authors concluded that serum ferritin is useful to identify NAFLD patients at risk for NASH and advanced fibrosis [33]. A recent report from Japan stated that the extent of serum ferritin elevations did not predict the stage of NAFLD although hyperferritinemia was common in NAFLD patients. The study is limited by a possible selection bias since only 19% of the study subjects had a histologic diagnosis [34].

In summary, a variety of proinflammatory biomarkers such as TNF-α, IL-6, CRP, and ferritin have been studied for their associations with NASH. Their performance in distinguishing NASH from simple steatosis is either fair or good but not excellent. Controversies of the association of each proinflammatory marker with NASH exist and well accepted cutoff values of each marker remain unknown.

### Constituents of extracellular matrix (ECM)

Liver fibrosis is one of the features of NASH [12]. The constituents of ECM are expected to be released into circulation during turnover of fibrosis in the liver. It is therefore reasonable using such markers to differentiate NASH from simple steatosis, especially those with significant liver fibrosis.

Yoneda et al. reported that marked elevation of serum hyaluronic acid (HA) and type IV collagen 7S domain, both ECM components, occurred in NASH patients with advanced fibrosis compared to those with mild fibrosis [24]. Serum HA levels were also markedly elevated in patients with NASH than with steatosis only. No cutoff value of either marker was provided in the study [24]. Another study reported that the best cutoff values to detect NASH using ROC analysis were ≥ 43 ng/mL for HA (AUROC 0.797) and ≥ 5 ng/mL for type IV collagen 7S domain (AUROC 0.828). The positive predictive value (PPV) for detecting NASH can be as high as 97.1% when both markers are greater than the cutoffs. In addition, the best cutoff values to detect severe fibrosis were ≥ 50 ng/mL for HA (AUROC 0.797) and ≥ 5 ng/mL for type IV collagen 7S domain (AUROC 0.817). The study reported that severe hepatic fibrosis is unlikely to be present if both markers are concomitantly less than the cutoffs [35]. In a study including only NASH patients, those with liver fibrosis had significantly higher serum HA and laminin levels than those without fibrosis. The best cutoff value was 148.8 ng/mL for HA (AUROC 0.975, sensitivity 97.5%, specificity 95.7%) and was 292.5 ng/mL for laminin (AUROC 0.789, sensitivity 73.9%, specificity 74.1%). The authors concluded that HA could be a predictive factor of the presence and stage of liver fibrosis in NASH. Laminin could be used to diagnose liver fibrosis but has no value in staging [36].

In contrast to the aforementioned findings, Malik et al. reported that there was no difference in levels of fibrosis markers such as HA, YKL-40, and tissue inhibitor of metalloproteinase 1 (TIMP1) between patients with NASH and with simple steatosis [37]. Similar to the previous study, a recent study reported that neither HA, TIMP1, YKL-40, nor collagen IV was associated with a histologic diagnosis of NASH. Pro-collagen III (PIIINP) was the only marker that was correlated with the total NAFLD activity score (NAS) and its constituent components. PIIINP was able to discriminate between patients with simple steatosis and those with NASH or advanced fibrosis with AUROC 0.85-0.87 [38].

In summary, ECM constituents seem to be decent markers for the detection of NASH although not without controversy. These markers are limited for routine use as they are not readily available in clinical laboratories. For better detection performances, these markers are often incorporated into diagnostic panels that are composed of several biomarkers. We will discuss these panels later in this review.

### Apoptosis markers

Apoptosis is a common mechanism of liver injury. Feldstein et al. [39] reported that the number of apoptotic cells was significantly increased in liver biopsy specimens from patients with NASH compared with those with simple steatosis and healthy controls. Immunohistochemistry demonstrated active caspases 3 and 7 in NASH specimens, confirming the occurrence of apoptosis in this condition. The apoptotic pathway is composed of two arms: the intrinsic pathway (initiated by cellular stress) and the extrinsic pathway (stimulated through a death receptor-mediated process). Both pathways are suspected to be involved in the pathogenesis of NASH [40]. In the final common step of apoptosis, the effector caspases (especially caspase 3 and caspase 7) are activated and cleave a number of different substrates inside the cell, including cytokeratin 18 (CK18), the major intermediate filament protein in the liver [41]. Plasma CK18 fragments were found to be markedly increased in patients with NASH (n=21) than those with simple steatosis (n=8) or normal controls (n=10) [median (interquartile range): 765.7 U/L (479.6-991.1), 202.4 U/L (160.4-258.2), 215.5 U/L (150.2-296.2), respectively; p < 0.001] [41]. A cutoff value of 395 U/L performed excellently for the diagnosis of NASH (AUROC 0.93, sensitivity 85.7, specificity 99.9%). For every 50 U/L increase in CK18 levels, the likelihood of having “definitive NASH” increased 86% (OR 1.86; 95% CI 1.23-2.82) [41]. By studying a larger cohort, the same group validated the significantly higher plasma CK18 fragments in patients with NASH (n=69) versus those without NASH (n=44) and borderline diagnosis (n=26) [median (25^th^, 75^th^ percentile), 335 (196, 511), 194 (151, 270), 200 (148, 284), respectively; p<0.001]. The AUROC for NASH diagnosis was estimated to be 0.83 [42]. A Greek study reported that fragmented CK18 levels had a good diagnostic accuracy for differentiating patients with NASH (n=30) from those with simple steatosis (n=28; AUROC 0.87). Levels of CK18 fragments ≥225 U/L, ≥ 250 U/L, or ≥ 300 U/L had sensitivity of 70%, 60%, or 53%; specificity of 82%, 93%, or 100%; PPV of 84%, 95%, or 100%; and negative predictive value (NPV) of 73%, 69%, or 67% for the diagnosis of NASH, respectively [43]. Using paired liver biopsies, serum CK18 fragment levels correlated with NAS in NAFLD patients [44]. The finding suggests that serum CK18 fragment levels can be used for monitoring NAFLD disease activity and treatment responses.

Two studies suggested that CK18 fragments may have a better performance for the diagnosis of NASH when combining with other tests [45,46]. First, a Turkish study reported that serum CK18 fragments had a fair performance in discriminating NASH from non-NASH (i.e., borderline NASH, simple steatosis, normal tissue) when using 121.6 U/L as the cutoff value (AUROC 0.787, sensitivity 60%, specificity 97.4). Using 243.8 U/L as the cutoff value, total serum CK18 levels had a better sensitivity and worse specificity than its apoptotic fragments for the diagnosis of NASH (AUROC 0.809, sensitivity 68.9, specificity 81.6). When combining CK18 with the liver attenuation on CT in Hounsfield units, both total CK18 and its apoptotic fragments had higher sensitivity (83.3% and 86.1%, respectively) for definitive NASH. The authors suggested that measurement of different forms of CK18 in combination with CT has greater diagnostic utility for the identification of patients with definitive NASH than the use of either test alone [45]. Second, a study from a group in Hong Kong reported a 2-step approach using CK18 fragments and fibroblast growth factor 21 (FGF21) further improved the accuracy in diagnosing NASH. In that study, at cutoffs of 203 and 670 U/L, serum CK18 fragments had a 71% NPV and 77% PPV to exclude and diagnose NASH. A 2-step approach measuring CK18 followed by FGF21 further improved the NPV to 74% and PPV to 82% [46].

Joka et al. [47] reported that both total serum CK18 and CK18 fragments could accurately differentiate healthy controls or simple steatosis from NASH and discriminate patients with different fibrosis stages from healthy controls. However, total CK18 levels had a better diagnostic performance and even differentiation between lower fibrosis stages as well as between healthy individuals and patients with simple steatosis than the apoptotic CK18 fragments [47].

In summary, NASH is associated with an increase in apoptotic activity in the liver. CK18 fragments are promising noninvasive biomarkers for the diagnosis of NASH. Based on the data from the paired biopsy study, serum levels of CK18 fragments can be possibly used to monitor liver disease activity and responses to treatment in patients with NASH.

### Diagnostic panels for NASH and advanced liver fibrosis

The diagnostic panels for NAFLD can be divided to two categories, one for the prediction of NASH and the other for the detection of liver fibrosis.

#### Panels for the detection of NASH

Different diagnostic panels for NASH are shown in Table 1. According to the HAIR scoring system, a weighted score of 2 or 3 is highly predictive of NASH. However, the study was confined to those with a BMI > 35 kg/m^2^ and the results may not be applicable to those with a lower BMI [48]. In the model reported by Palekar et al., patients with three or more of the following six characteristics are more likely to have NASH than simple steatosis: female gender, age ≥ 50 years, BMI ≥ 30 kg/m^2^, aspartate aminotransferase (AST) ≥ 45 U/L, AST/ALT ratio ≥ 0.8, and HA ≥55 mcg/L [49]. Combining 13 clinical and biochemical parameters, the NashTest only had a fair performance (AUROC 0.78) but a very good specificity (94%) for predicting NASH [50]. Comparing with the NashTest, Gholam et al. was able to predict NASH in a severely obese cohort (BMI ≥ 40 kg/m^2^) by simply using two markers, AST and the presence of diabetes (AUROC 0.82) [51].

**Table 1:** Accuracy of diagnostic panels for NASH

**Reference**	**Biomarkers**	**N**	**AUROC**	**Cutoff value**	**Se (%)**	**Sp (%)**	**PPV (%)**	**NPV (%)**
Dixon [48]	HAIR score	105	0.90	score>2	80	89	n/a	n/a
	(hypertension, ALT>40 U/L, IR index>5)							
Palekar [49]	age≥50 years, female gender, AST≥45 U/L,	80	0.76	score≥3	73.7	65.7	68.2	71.4
	BMI≥30 kg/m^2^, AAR ratio≥0.8, HA≥55 mcg/L							
Poynard [50]	NashTest	257	0.78	n/a	33	94	66	81
	(age, sex, height, weight, triglyceride, GGT,							
	cholesterol, alpha2macroglobulin, AST, ALT,							
	apolipoprotein A1, haptoglobin, total bilirubin)							
Gholam [51]	AST, presence of diabetes	97	0.82	8.22	76	66	n/a	n/a
Campos [52]	NASH Clinical Scoring System for Morbid	186	0.80	0-2			7	93
	obesity (hypertension, type 2 diabetes,			3-4			27	73
	AST≥27 U/L, ALT ≥27 U/L, sleep apnea,			5			59	41
	non-black race)			6-7			93	7
Ulitsky [53]	diabetes, ALT >40 U/L, triglyceride >150	253	0.76	0-1			10.6	89.4
	mg/dL, sleep apnea			2-3			24.7	75.3
				4			60	40
				5			75	25
Shimada [54]	adiponectin≤4 mcg/mL, HOMA-IR≥3,	85	n/a	all 3 +ive	94	74	94	74
	type IV collagen 7S≥5 ng/mL							
Tarek [56]	CK18 fragments, sFas	95	0.93	−0.5509	88	89	n/a	n/a
Younossi [57]	NASH Diagnostics	101	0.908	0.2772	94.5	70.2	60	97.1
	(cleaved and intact CK18 levels,							
	serum adiponectin and resistin levels)							
Sookoain [58]	BMI, waist circumference, ALT, AST, AP,	101	0.795	n/a	n/a	n/a	n/a	n/a
	GGT, HOMA, CRP, sICAM-1							
Sumida [59]	NAFIC score	177^†^	0.851^†^	1	94	48	31	86
	[serum ferritin (≥200 ng/mL for female,			2	66	91	90	67
	≥300 ng/mL for male), fasting insulin ≥10	442^¥^	0.782^¥^	1	88	43	66	75
	μU/mL, type IV collagen ≥5 ng/mL]			2	60	87	85	64
Younossi [60]	NAFLD Diagnostic Panel	79	0.81	0.221	91.2	47.4	60.8	85.7
	(diabetes, gender, BMI, triglycerides,			0.3641	79.4	73.7	73	80
	apoptotic and necrotic CK18 fragments)			0.6183	44.1	92.1	83.3	64.8

AUROC, area under receiver operator characteristic curve; Se, sensitivity; Sp, specificity; PPV, positive predictive value; NPV, negative predictive value; ALT, alanine aminotransferase; IR, insulin resistance; AST, aspartate aminotransferase; BMI, body mass index; AAR, AST/ALT ratio; HA, hyaluronic acid; GGT, gamma-glutamyl transpeptidase; HOMA-IR, homeostasis model assessment of insulin resistance; AP, alkaline phosphatase; CRP, C-reactive protein; n/a, not available.

^†^Estimation group; ^¥^Validation group.

Obstructive sleep apnea has been shown to improve diagnostic accuracy of the panels for NASH [52,53]. The NASH Clinical Scoring System for Morbid Obesity is consisted of six components including hypertension, type 2 diabetes, AST ≥ 27 U/L, ALT ≥ 27 U/L, sleep apnea, and non-black race. Two points are assigned to non-black race and one point is assigned to the rest of the five components. The probability of NASH is determined based on the summed points. A cohort of morbidly obese patients (n=186) was divided into four categories based on the probability of NASH (low, intermediate, high, and very high). Both the PPV of the very high-risk category and the NPV of the low-risk category for NASH were 93% and the AUROC was 0.8 [52]. A simplified scoring model based on only four variables including type 2 diabetes, ALT > 40 U/L, triglyceride > 150 mg/dL, and obstructive sleep apnea was reported by Ulitsky et al. [53]. Similar to the design of the previous study, two points are assigned to diabetes and one point is assigned to the other three variables. A cohort of morbidly obese patients (n=235) was divided into four groups based on the risk of NASH determined by the summed points (low, intermediate, high, and very high). The model performed similarly to the previous study (AUROC 0.76 vs. 0.8) [52,53]. Both studies suggested that a liver biopsy should be recommended for morbidly obese individuals who are in the high and very high-risk categories for NASH. On the other hand, a liver biopsy can be delayed or avoided for individuals with a low risk of NASH [52,53].

Shimada et al. reported that approximately 90% of the patients with early-stage NASH can be predicted by a combined evaluation of the serum adiponectin level, homeostasis model assessment of insulin resistance (HOMA-IR), and serum type IV collagen 7S level [54]. Both adiponectin and resistin are adipokines mainly produced by adipose tissues and both are involved in insulin resistance. Hypoadiponectinemia is commonly seen in patients with NAFLD [55]. Tarek et al. developed a panel that consisted of 2 apoptosis markers (CK18 fragments and soluble Fas) for NASH [56]. In the initial cohort that included patients suspicious for having NASH, the panel performed quite well with an AUROC 0.93. In the validation cohort that included morbidly obese patients only, the panel still had a fair performance with an AUROC 0.79. The high AUROC in the validation group indicated that this panel provides reproducible results even for a highly distinct group of patients [56].

Given the complexity of the pathogenesis of NASH, it is likely that multiple pathways may play critical roles in the development of the disease. Younossi et al. therefore developed the NASH diagnostics model that includes two apoptosis markers (apoptosis- and necrosis-derived CK18 fragments) and two adipokines (adiponectin and resistin) [57]. If the model were used to minimize the use of liver biopsies at the threshold of 0.2085, 79.2% of the study patients could avoid the invasive procedure. Again, the study subjects were morbidly obese and the study results may not be generalized to non-obese population [57]. Sookoian et al. designed a diagnostic panel based on a composite index using nine markers derived from both clinical and routine laboratory data [58]. Considering a model patient with all the positive tests, the post-test probability for NASH would be 99.5%. In the same way, considering a model with all negative tests, the post-test probability for NASH would be negligible (0.3%). Nevertheless, the performance of the model was only fair (AUROC 0.795) [58].

Based on the head-to-head comparison studies, two panels were reported outperforming several other existed panels previously mentioned. First, the NAFIC scoring system using the following markers: serum ferritin (≥ 200 ng/mL for female; ≥ 300 ng/mL for male), fasting insulin (≥10 μU/mL), and type IV collagen 7S (≥ 5 ng/mL), outperformed the HAIR scoring system [48] and the scoring system developed by Palekar et al. [49] and Gholam et al. [51] for differentiating NASH from simple steatosis [59]. The study results, however, may not be adaptable for NAFLD patients of other races since all participants of the study were Japanese. Second, the NAFLD Diagnostic Panel using five markers: diabetes, gender, BMI, triglycerides, apoptotic and necrotic CK18 fragments, had a better performance than the NASH Diagnostics for predicting histologic NASH [60]. The study participants in both studies had similar BMIs and both panels included CK18 fragments as a marker [57,60].

#### Panels for the detection of advanced liver fibrosis

Several diagnostic panels have been developed for the prediction of significant liver fibrosis (Table 2). Angulo et al. suggested that NASH patients who are older (≥ 45 years), obese (BMI >31.1 kg/m^2^ for male, > 32.3 kg/m^2^ for female), and suffer from diabetes mellitus are at greatest risk of progressing to cirrhosis with its consequent complications. They also proposed that patients with those characteristics should be greatly encouraged to undergo liver biopsy so intervention can be offered [61]. The BAAT scoring system is calculated as the sum of the following parameters: age at liver biopsy (≥ 50 years = 1; < 50 years = 0), BMI (≥28 kg/m^2^ = 1; < 28 kg/m^2^ = 0), triglycerides (≥1.7 mmol/L = 1; <1.7 mmol/L = 0), and ALT (≥ 2 times of normal = 1; < 2 times of normal = 0). A score of 0 or 1 had 100% NPV for the diagnosis of septal fibrosis and cirrhosis [62]. The European Liver Fibrosis Group studied 1021 subjects who had a variety of chronic liver diseases and came up with the Original European Liver Fibrosis panel (OELF). The panel consists of age and three serum markers, HA, TIMP1, and PIIINP. For NAFLD, fibrosis stage 3 or 4 was detectable using a threshold value of 0.375 with a sensitivity of 89%, specificity of 96%, PPV of 80%, and NPV of 98% [63]. The Enhanced Liver Fibrosis panel (ELF) differs from the OELF by simply removing age from the panel. Despite that change, the ELF had an excellent AUROC of 0.90 for distinguishing severe fibrosis (F0-2 vs. F3-4), good performance (AUROC 0.82) for moderate fibrosis (F0-1 vs. F2-4), and fair performance (AUROC 0.76) for no fibrosis (F0 vs. F1-4). By using the ELF, Guha et al. reported that 82% of liver biopsies in their study could be potentially avoided for the diagnosis of severe fibrosis [64].

**Table 2:** Accuracy of diagnostic panels for advanced liver fibrosis

**Reference**	**Biomarkers**	**N**	**AUROC**	**Cutoff value**	**Se (%)**	**Sp (%)**	**PPV (%)**	**NPV (%)**
Angulo [61]	age≥45 years, obesity (BMI>31.1 for male,	144	n/a	n/a	n/a	n/a	n/a	n/a
	>32.3 for female), diabetes, AAR>1							
Ratziu [62]	BAAT score	93	0.84	0	100	11	33	100
	(BMI≥28 kg/m^2^, age≥50 years, ALT≥2xULN,			1	100	47	45	100
	triglycerides≥1.7 mmol/L)			2	71	80	61	86
				3	14	100	100	73
				4	0	100	0	70
Rosenberg	Original European Liver Fibrosis panel	61	0.87	0.375	89	96	80	98
[63]	(age, HA, TIMP1, PIIINP)			0.462	78	98	87	96
Guha [64]	Enhanced Liver Fibrosis panel	192	0.90	0.3576	80	90	71	94
	(HA, TIMP1, PIIINP)							
Gholam [51]	ALT, HbA1C	97	0.90	6.6	83	82	n/a	n/a
Ratziu [65]	FibroTest	267	0.81	0.3	77	77	54	90
	(α2 macroglobulin, haptoglobin, GGT,			0.7	15	98	73	76
	Total bilirubin, apolipoprotein A1)							
Angulo [66]	NAFLD Fibrosis Score	733	0.84	<−1.455^†^	82	77	56	93
	(age, hyperglycemia, BMI, platelet count,			>0.676^†^	51	98	90	85
	albumin, AAR)			<−1.455^¥^	77	71	52	88
				>0.676^¥^	43	96	82	80
Harrison [67]	BARD score	827	0.81	2-4			43	96
	(BMI≥28 kg/m^2^, AAR≥0.8, diabetes)							
Cales [68]	Fibrometer NAFLD	235	0.943		78.5	95.9	87.9	92.1
	(glucose, AST, ferritin, ALT, body weight,							
	age)							
Shah [69]	FIB4 index	541	0.802	<1.30	74	71	43	90
	(age, ALT, AST, platelet count)			>2.67	33	98	80	83
Sumida [59]	NAFIC score	619	0.834	0	95	33	32	96
	[serum ferritin (≥200 ng/mL for female,			2-4	84	74	52	93
	≥300 ng/mL for male), fasting insulin≥10							
	μU/mL, type IV collagen 7S≥5 ng/mL]							
Younossi [60]	NAFLD Diagnostic Panel	79	0.80	0.2188	90.91	43.59	57.7	85
	(diabetes, gender, BMI, triglycerides,			0.4242	60.61	71.79	64.5	68.3
	apoptotic and necrotic CK18 fragments)			0.5689	51.52	89.74	81	68.6

AUROC, area under receiver operator characteristic curve; Se, sensitivity; Sp, specificity; PPV, positive predictive value; NPV, negative predictive value; ALT, alanine aminotransferase; AST, aspartate aminotransferase; BMI, body mass index; AAR, AST/ALT ratio; HA, hyaluronic acid; GGT, gamma-glutamyl transpeptidase; TIMP1, tissue inhibitor of metalloproteinase 1; PIIINP, amino-terminal propeptide of type III collagen; ULN, upper limit of normal; n/a, not available.

^†^Estimation group; ^¥^Validation group.

The commercially available FibroTest incorporates five serum markers, α2 macroglobulin, haptoglobin, apolipoprotein A1, gamma-glutamyl transpeptidase (GGT), and total bilirubin. It has a good performance for detecting F2 (AUROC 0.81) and F3 (AUROC 0.88) fibrosis. The probability of cirrhosis is very low when the test score is below 0.3. On the other hand, the patient should be managed as a patient with cirrhosis when the score is 0.7 or higher [65]. By simply using ALT and glycosylated hemoglobin A1C, Gholam et al. reported an excellent performance (AUROC 0.90) of their model for detection of liver fibrosis in severely obese subjects (BMI ≥ 40 kg/m^2^) [51].

The NAFLD Fibrosis Score (NFS) was developed from a large cohort (n=773) and is calculated from six variables: age, hyperglycemia, BMI, platelet count, albumin, and AST/ALT ratio. By applying the low cutoff score (−1.455), advanced fibrosis could be excluded with high accuracy (NPV of 93% and 88% in the estimation and validation groups, respectively). By applying the high cutoff score (0.676), the presence of advanced fibrosis could be diagnosed with high accuracy (PPV of 90% and 82% in the estimation and validation groups, respectively). By using this model, Angulo et al. reported that a liver biopsy would have been avoided in 75% of the study cohort and with a correct prediction of 90% [66]. Since being published, the NFS has been directly compared with several other models. For example, the easily calculated BARD score was reported to be at least equivalent to the more complex NFS in excluding patients with advanced fibrosis [67]. Depending on the presence of the following variables, BMI ≥ 28 kg/m^2^ (1 point), AST/ALT ratio ≥ 0.8 (2 points), and diabetes (1 point), the BARD score ranges from 0 to 4. A score of 2–4 was associated with an OR for advanced fibrosis of 17 (95% CI 9.2-31.9) and a NPV of 96%. The BARD score seems to be more accurate at predicting advanced fibrosis in non-diabetics compared with diabetic patients [67]. The Fibrometer NAFLD, which is computed from seven easily obtainable variables (glucose, AST, ferritin, platelet, ALT, body weight and age), was reported to be more accurate than the NFS [68]. The FIB4 index was reported outperforming seven other noninvasive markers of fibrosis in patients with NAFLD, including the NFS, based on the analysis of a multi-center cohort [69]. A FIB4 index ≥ 2.67 had an 80% PPV and a FIB4 index ≤1.30 had a 90% NPV of significant liver fibrosis [69]. As mentioned previously, the NAFIC score was developed based on a large Japanese cohort [59]. After a direct comparison, it was concluded that the easily determined NAFIC score was at least equivalent to the more complex NFS. The authors further suggested that liver biopsy can be avoided in NAFLD patients with a NAFIC score of 0 or 1 because they are likely to have NAFLD without advanced fibrosis. In contrast, liver biopsy should be recommended in NAFLD patients with an NAFIC score of ≥ 2 to assess the extent of hepatic fibrosis and predict prognosis [59]. Finally, the NAFLD Diagnostic Panel was directly compared with the NFS in a recent study with a much smaller cohort [60]. For predicting either any degree of fibrosis or advanced fibrosis, the NAFLD Diagnostic Panel performed significantly better than that of the combined ELF and NFS model and ELF alone or NFS alone (any fibrosis and advanced fibrosis AUROC, 0.80 and 0.81, 0.78 and 0.63, 0.76 and 0.65, 0.71 and 0.59, respectively) [60].

In summary, several diagnostic panels have been developed for the prediction of NASH and significant fibrosis in patients with NAFLD. Each panel is unique for its biomarker composition. The cohorts in different studies were heterogeneous in characteristics and sometimes highly selected, which may limit their utility in different populations. The recent published practice guideline recommends the NFS as a clinically useful tool for identifying NAFLD patients with higher likelihood of having bridging fibrosis and/or cirrhosis. In addition, the presence of MetS and the NFS may be used for identifying patients who are at risk for steatohepatitis and advanced fibrosis [70].

### Biomarkers for NAFLD defined by proteomic studies

Using the promising proteomics, a few studies have been conducted to identify potential biomarkers for NAFLD. By studying bariatric surgery patients (n=98), Younossi et al. first found 12 protein peaks with significant differential expression when sera from NAFLD groups (n=91) were compared with obese controls (n=7) [71]. A prospective study identified three protein peaks, which were related to different hemoglobin subunits, progressively increased in intensity from patients who were obese but without liver lesions (n=24) to those with steatosis (n=32) and NASH (n=24), but returned to near normal value after patients had lost weight [72]. Bell et al. reported the expression levels of 55 and 15 proteins changed significantly between the simple steatosis (n=24) and NASH with advanced fibrosis (n=22) groups and the NASH (n=23) and NASH with advanced fibrosis groups, respectively. There were, however, no significant differences observed between the simple steatosis and NASH groups, suggesting that systemic markers of fatty liver and NASH may not be present in serum from patients with mild disease [73]. Those proteins were found to be involved in immune regulation and inflammation, coagulation, cellular and extracellular matrix structure and function, and roles as carrier proteins in the blood [73].

In addition to sera, proteomics have also been used in other tissues to discover biomarkers for NAFLD. By studying liver specimens from severely obese patients, Charlton et al. discovered nine proteins with differential expression between study groups [74]. Lumican, a 40-kDa keratin sulfate proteoglycan that regulates collagen fibril assembly and activates transforming growth factor-beta and smooth muscle actin, was overexpressed in a progressive manner in NASH-mild versus simple steatosis (124%, p < 0.001), NASH-progressive versus NASH-mild (156%, p < 0.001), and NASH-progressive versus obese normal (178%, p < 0.001). Fatty acid binding protein-1, which is protective against the detergent effects of excess fatty acids, was underexpressed in NASH-mild versus simple steatosis (73%, p < 0.001), NASH-progressive versus NASH-mild (81%, p < 0.001), and NASH-progressive versus obese normal (59%, p < 0.001) [74]. Another study analyzed liver samples from severely obese patients and identified two candidate markers, CPS1 and GRP78, which were down-regulated in NASH versus normal control groups [75]. CPS1 and GRP78 were also confirmed to be serum candidate markers of NAFLD. Their serum concentrations decreased gradually from control subjects to steatosis and NASH patients [75]. By studying visceral adipose tissue, Younossi et al. reported that a model based on a combination of clinical and phosphoproteomic parameters had a good performance in predicting NASH (AUROC 0.86, sensitivity 81.3%, specificity 87%) [76].

In contrast to the previous reports, Ulukaya et al. reported that serum proteomic pattern analysis did not help to distinguish NASH from simple steatosis in patients with NAFLD [77]. Compared to the prior studies, the study subjects had a much lower BMI, which may explain the disparity.

In summary, proteomics is a promising technology that can screen a large number of proteins in a high throughput fashion. The use of proteomics in discovery of biomarkers for NAFLD is still in early stages. Most of the data mentioned in this section were derived from severely obese subjects who were mainly Caucasians. The findings need to be validated in other population in the future.

## Conclusion

Several biomarkers that are associated with inflammation, ECM, and apoptosis have been individually studied for their performances in distinguishing NASH from simple steatosis. Routine use of individual marker is limited by the controversial published results and commercial unavailability. Several panels of biomarkers in variable combination have been developed for the detection of either NASH and/or advanced liver fibrosis. Most of the panels have not been validated in longitudinal studies. The study cohorts are heterogeneous in characteristics and sometimes highly selected. Future studies should be ideally designed as population-based, prospective, and longitudinal in order to eliminate selection and referral biases and to validate the results derived from cross-sectional studies.

## Competing interests

The authors declare that they have no competing interests.

## Authors’ contributions

SP, NT and JP drafted the manuscript. All authors read and approved the final manuscript.
